# The Death Rate from Nephritis in 140 Cities in the United States, for 1898

**Published:** 1900-01

**Authors:** C. H. Wilkinson

**Affiliations:** Health Physician of Galveston, Texas


### TEXAS MEDICAL JOURNAL.

ESTABLISHED JULY, 1885.

PUBLISHED MONTHLY.—SUBSCRIPTION $1.00 A YEAR.

VOL. XV. AUSTIN, JANUARY, 1900. No. 7.

### Original Contributions.

For Texas Medical Journal.

### The Death Rate From Nephritis in 140 Cities in the United States, for 1898.

BY C. H. WILKINSON, M. D.,
HEALTH PHYSICIAN OF GALVESTON, TEXAS.

In presenting the following table of deaths from nephritis in 140 of the principal cities of the United States for the year 1898, it is my desire to call the attention of the profession to the universality, to the steady and rapid increase throughout the country, and to the favored localities of this disease. The table, which has been compiled from the "Bulletin of the Department of Labor," for September, 1899, is authentic, and should prove valuable to every sanitarian who desires to keep abreast with one of the most insidious as well as one of the most formidable general diseases of the day.

In this report an opportunity for studying the causation of nephritis may be afforded the profession, inasmuch as the favored localities of the disease can be taken in at a glance, and cherished theories as to its nature may be exploded or encouraged.

No elaborate effort to eliminate the cause of this disease will be indulged in by the writer; the salient features as stated above, to-wit, its geographical distribution, and rates to population in American cities alone being given.

The careful study of the table may, however, aid others in the search, and should any practical value result from its consideration, the writer will be well rewarded for his labors in preparing it.

As is well known, many and varied sources have been assigned to the causation of nephritis. Air, water, climate, food, habits and a number of other factors have been reckoned on as the primary cause of this disease, and each has had its champions, yet the origin of nephritis remains today about as far from satisfactory solution as it was some twenty years ago.

Malaria, as might be expected, perhaps stands at the head of the list in point of causation; for a while, alcohol was cited as the true and only origin of the evil. The worry of occupation; the drinking of artesian waters; the accumulation of uric acid in the system; the result of skin diseases, saline atmosphere, and the sudden checking of perspiration have all had their votaries, and yet the true cause seems as mysterious as ever.

To the writer, it appears more probable that the cause of Bright's disease is due to a bacillus. All our important, general diseases depend upon such cause; notably is this true of typhoid fever and consumption, diseases which, a few years ago, were attributed to many of the causes charged up to nephritis today.

In fact, from a geographical standpoint, these last named diseases would seem related, for wherever phthisis prevails, to a great extent nephritis also soars high in the column of demises. A priori reason would place nephritis in the ranks of bacilliary diseases, and if such were the case, then this disorder stands alone as a remarkable exception to the general rule.

In proof of the correctness of the foregoing view, it may be stated that Mannaberg has detected a micro-organism in the urine of patients suffering with Bright's disease, "whose culture was unlike that of any other organism known," and which produced nephritic symptoms in animals experimented on.

A glance at the table shows the alarming ratio of this disease in Charleston, South Carolina, to the number of the inhabitants of that city. On the other hand, nephritis is scarcely yet prevalent in Butte, Montana. The cities of Covington, Kentucky, and Cincinnati, Ohio, are merely separated by the Ohio river, yet note the different death ratios from the disease in these two cities. Galveston is entirely surrounded by a saline atmosphere, and her citizens almost exclusively drink artesian water. On the other hand, the people of New Orleans are subjected to neither of these features, yet the latter has a death rate much larger that the former from nephritic diseases. Galveston is practically free from miasmatic diseases, yet nephritis claims one victim in every 1200 of her inhabitants, and ranks second on her mortuary record, consumption being first.

Contrasts might be drawn in many other instances in disproof of some pet theory of cause, but enough have been recited to enable the searcher after truth to carry out the investigation to some definite conclusion.

These facts, however, would seem to be established by the report —that many of the agencies heretofore believed to be important factors in the development of nephritis cut no figure in its causation whatever, and secondly, that the nearer we approach the level of the sea, the higher does the death rate in this disease appear. Per contra, the farther away we proceed from the ocean level, the lower does the deaths to population become. In fact, the elevated cities of the country, and notably so is this of Denver, Colorado, and Butte, Montana, seem to be but seldom visited by nephritis.

A final fact the writer wishes to impress upon the reader is that Bright's disease is not confined to southern territory as some of the actuaries of large insurance companies might infer, since New York City, Boston, Chicago, and particularly Washington, and Albany display as high a death rate in this disease as many of the cities lying further south.

The Department of Labor has undertaken a most valuable enterprise in thus collecting and publishing the death rates of our American cities, and the wish is expressed that it may continue its laudable work for many years. By doing so a tab can be kept upon this and other diseases, and an opportunity be given for our citizens to select that location best fitted for the prevention of such maladies.

| Names of cities. | Estimated population. | Disease nephritis. | Ratio to population. | Names of cities. | Estimated population. | Disease nephritis. | Ratio to population. |
|---|---|---|---|---|---|---|---|
| | | | one in | | | | one in |
| Akron, Ohio | 40,000 | 28 | 1428 | Malden, Mass | 32,500 | 30 | 1083 |
| Albany, N. Y. | 100,000 | 161 | 621 | Manchester, N. H. | 55,000 | 32 | 1718 |
| Alleghany, Pa. | 125,000 | 41 | 3148 | Memphis, Tenn | 75,000 | 49 | 1530 |
| Allentown, Pa | 35,000 | 18 | 1944 | Milwaukee, Wis | 280,000 | 106 | 2641 |
| Altoona, Pa. | 40,000 | 11 | 3633 | Minneapolis, Minn | 225,602 | 95 | 2374 |
| Atlanta, Ga | 96,500 | 45 | 2144 | Mobile, Ala | 38,000 | 68 | 558 |
| Auburn, N. Y. | 32,000 | 16 | 2000 | Nashville, Tenn | 90,000 | 49 | 1837 |
| Augusta, Ga | 50,000 | 27 | 1852 | Newark, N. J | 275,000 | 231 | 1190 |
| Baltimore, Md | 541,000 | 534 | 1013 | New Bedford, Mass | 56,000 | 53 | 1056 |
| Bay City, Mich | 38,000 | 10 | 3800 | New Haven, Conn | 11(,000 | 109 | 1008 |
| Binghampton, N. Y. | 45,000 | 26 | 1731 | New Orleans, La | 285,000 | 369 | 752 |
| Birmingham, Ala | 37,500 | 14 | 2678 | Newport, Ky | 31,000 | 14 | 3350 |
| Boston, Mass | 582,493 | 398 | 1463 | New York, N. Y | 3,500,000 | 4,687 | 746 |
| Bridgeport, Conn | 70,000 | 80 | 875 | Norfolk, Va | 65,000 | 61 | 1065 |
| Brockton, Mass | 37,278 | 11 | 3389 | Oakland, Cal | 75,000 | 29 | 2586 |
| Buffalo, N. Y | 400,000 | 190 | 2105 | Omaha, Neb | 158,000 | 30 | 5266 |
| Butte, Mont | 50,000 | 8 | 6250 | Oshkosh, Wis | 30,000 | 7 | 4485 |
| Cambridge, Mass | 90,000 | 65 | 1384 | Paterson, N. J | 110,500 | 48 | 2303 |
| Camden, N. J | 70,000 | 56 | 1250 | ~~Pawtucket, R. I~~ | 35,000 | 36 | 972 |
| Canton, Ohio | 114,200 | 7 | 6314 | Peoria, Ill | 52,000 | 19 | 2737 |
| Charleston, S. C. | 68,000 | 176 | 386 | Philadelphia, Pa | 1,240,766 | 1134 | 1092 |
| Chattanooga, Tenn | 30,000 | 15 | 2000 | Pittsburg, Pa | 298,772 | 153 | 1952 |
| Chelsea, Mass | 33,468 | 18 | 1859 | Portland, Maine | 41,500 | 41 | 1012 |
| Chicago, Ill | 1,850,000 | 1,048 | 1765 | Portland, Oregon | 92,413 | 20 | 4620 |
| Cincinnati, Ohio | 415,000 | 243 | 1707 | Providence, R. I | 166,000 | 193 | 850 |
| Cleveland, Ohio | 380,000 | 161 | 2360 | Pueblo, Colo | 43,645 | 13 | 3357 |
| Columbus, Ohio | 140,000 | 84 | 1666 | Quincy, Ill | 42,000 | 24 | 1791 |
| Covington, Ky | 55,000 | 57 | 965 | Reading, Pa | 76,000 | 34 | 2235 |
| Dallas, Texas | 50,000 | 24 | 2083 | Richmond, Va | 105,000 | 51 | 2058 |
| Davenport, Iowa | 40,000 | 19 | 2105 | Rochester, N. Y | 175,000 | 142 | 1132 |
| Dayton, Ohio | 85,000 | 40 | 2125 | Rockford, Ill | 33,000 | 3 | 11000 |
| Denver, Colo | 170,000 | 88 | 1932 | Sacramento, Cal | 34,765 | 9 | 3862 |
| Des Moines, Iowa | 70,000 | 18 | 3889 | Saginaw, Mich | 60,000 | 23 | 2601 |
| Detroit, Mich | 350,000 | 146 | 2397 | St. Joseph, Mo | 65,000 | 15 | 5000 |
| Dubuque, Iowa | 45,000 | 10 | 4500 | St. Louis, Mo | 623,000 | 398 | 1681 |
| Duluth, Minn | 60,000 | 24 | 2500 | St. Paul, Minn | 215,582 | 42 | 5133 |
| Elizabeth, N. J | 50,000 | 53 | 941 | Salem, Mass | 36,000 | 27 | 1333 |
| Elmira, N. Y | 42,000 | 32 | 1812 | Salt Lake City, Utah | 70,000 | 21 | 3333 |
| Erie, Pa | 60,000 | 23 | 2608 | San Antonio, Texas | 00 | 00 | 00 |
| Evansville, Ind | 67,000 | 37 | 1811 | San Francisco, Cal | 360,000 | 289 | 1245 |
| Fall River, Mass | 97,517 | 38 | 2566 | Savannah, Ga | 65,000 | 39 | 1666 |
| Fort Wayne, Ind | 50,000 | 36 | 1388 | Scranton, Pa | 105,000 | 41 | 2561 |
| Fort Worth, Texas | 35,000 | 00 | 00 | Seattle, Wash | 75,000 | 11 | 6818 |
| Galveston, Texas | 60,000 | 50 | 1200 | Sioux City, Iowa | 35,000 | 10 | 3500 |
| Gloucester, Mass | 30,500 | 12 | 2541 | Somerville, Mass | 60,000 | 30 | 2000 |
| Grand Rapids, Mich | 99,000 | 31 | 2193 | South Bend, Ind | 32,000 | 8 | 4000 |
| Harrisburg, Pa | 50,000 | 22 | 2272 | Spokane, Wash | 45,000 | 18 | 2500 |
| Hartford, Conn | 77,000 | 69 | 1116 | Springfield, Ill | 43,000 | 26 | 1715 |
| Haverhill, Mass | 36,100 | 21 | 2719 | Springfield, Mass | 57,676 | 87 | 663 |
| Hoboken, N. J | 64,463 | 62 | 1039 | Springfield, Mo | 30,000 | 00 | 00 |
| Holyoke, Mass | 44,982 | 25 | 1799 | Springfield, Ohio | 40,000 | 25 | 1600 |
| Houston, Texas | 60,000 | 16 | 3750 | Superior, Wis | 35,000 | 2 | 17500 |
| Indianapolis, Ind | 200,000 | 83 | 2409 | Syracuse, N. Y | 130,000 | 39 | 3333 |
| Jersey City, N. J | 195,847 | 150 | 1305 | Tacoma, Wash | 50,000 | 11 | 4545 |
| Johnstown, Pa | 31,000 | 11 | 2818 | Taunton, Mass | 30,000 | 20 | 1500 |
| Joliet, Ill | 30,000 | 9 | 3333 | Terre Haute, Ind | 40,000 | 4 | 10000 |
| Kansas City, Kans | 48,000 | 9 | 5333 | Toledo, Ohio | 142,000 | 45 | 3155 |
| Kansas City, Mo | 200,000 | 44 | 4545 | Topeka, Kans | 35,000 | 9 | 3889 |
| Knoxville, Tenn | 40,000 | 8 | 5000 | Trenton, N. J | 73,000 | 26 | 2027 |
| La Crosse, Wis | 32,000 | 9 | 3711 | Troy, N. Y | 67,000 | 45 | 1488 |
| Lancaster, Pa | 43,160 | 25 | 1726 | Utica, N. Y | 60,000 | 46 | 1304 |
| Lawrence, Mass | 57,263 | 21 | 2727 | Washington, D. C | 387,462 | 301 | 955 |
| Lincoln, Neb | 60,000 | 18 | 3333 | Waterbury, Conn | 41,000 | 31 | 1322 |
| Little Rock, Ark | 40,000 | 18 | 2222 | Wheeling, W. Va | 38,000 | 24 | 1583 |
| Los Angelos, Cal | 110,000 | 81 | ~~2586~~ | Wilkesbarre, Pa | 50,000 | 22 | 2272 |
| Louisville, Ky | 225,000 | 87 | 2586 | Williamsport, Pa | 32,000 | 19 | 1684 |
| Lowell, Mass | 88,641 | 91 | 963 | Wilmington, Del | 72,000 | 38 | 1846 |
| Lynn, Mass | 67,600 | 42 | 1595 | Worcester, Mass | 105,000 | 87 | 1207 |
| McKeesport, Pa | 32,000 | 4 | 8000 | Yonkers, N. Y | 45,000 | 39 | 1154 |
| Macon, Ga | 30,000 | 5 | 6000 | Youngstown, Ohio | 52,000 | 13 | 4004 |

